# GABA Type A receptors expressed in triple negative breast cancer cells mediate chloride ion flux

**DOI:** 10.3389/fphar.2024.1449256

**Published:** 2024-10-14

**Authors:** J Bundy, Y Ahmed, S Weller, J Nguyen, J Shaw, I Mercier, A Suryanarayanan

**Affiliations:** Department of Pharmaceutical Sciences, Philadelphia College of Pharmacy, Saint Joseph’s University, Pharmacology and Toxicology Center, Philadelphia, PA, United States

**Keywords:** GABA type A receptor, GABA beta3, ligand gated chloride channel, TNBC (triple negative breast cancer), ion flux, bicuculline

## Abstract

Triple negative breast cancer (TNBC) is known for its heterogeneous nature and aggressive onset, limited unresponsiveness to hormone therapies and immunotherapy as well as high likelihood of metastasis and recurrence. Since no targeted standard treatment options are available for TNBC, novel and effective therapeutic targets are urgently needed. Ion channels have emerged as possible novel therapeutic candidates for cancer therapy. We previously showed that GABA_A_ β3 subunit are expressed at higher levels in TNBC cell lines than non-tumorigenic MCF10A cells. GABA_A_ β3 subunit knockdown causes cell cycle arrest in TNBC cell lines via decreased cyclin D1 and increased p21 expression. However, it is not known if the upregulated GABA_A_R express at the cell-surface in TNBC and mediate Cl^−^ flux. Cl^−^ ions are known to play a role in cell-cycle progression in other cancers such as gastric cancer. Here, using surface biotinylation and (N-(Ethoxycarbonylmethyl)-6-Methoxyquinolinium Bromide) MQAE-dye based fluorescence quenching, we show that upregulated GABA_A_R are on the cell-surface in TNBC cell lines and mediate significantly higher chloride (Cl^−^) flux as compared to non-tumorigenic MCF10A cells. Moreover, this GABA_A_R mediated Cl^−^ flux can be modulated by pharmacological agents and is decreased in TNBC cells with GABA_A_ β3 subunit knockdown. Further, treatment of TNBC cells with bicuculline, a GABA_A_R antagonist reduced cell viability in TNBC cells Overall, these results point to an unexplored role of GABA_A_R mediated Cl^−^ flux in TNBC.

## Introduction

Triple negative breast cancer (TNBC) is a subtype of breast cancer that lacks estrogen receptor (ER), progesterone receptor (PR), and HER2 expression (Lee et al., 2020). The majority of TNBC shows the expression of basal markers along with a smaller fraction that lacks the expression of basal markers and is called non-basal-like (Sahoo et al., 2024). Surgery, radiotherapy, chemotherapy, and immunotherapy are the treatment options for TNBC patients. However, TNBC treatment options are limited due to its unresponsiveness to hormone therapy and anti-HER2 therapy (Zagami and Carey, 2022; Agostinetto et al., 2022). TNBC is recognized for its heterogeneity, extremely aggressive onset as well as high occurrence of metastasis, highlighting an urgent need for targeted therapies.

Recently, ion channels have emerged as a potential target in cancer therapy (Capatina et al., 2022). Ion channels are imperative for maintaining the control of membrane potential, cell signaling, and the movement of ions necessary for cellular functions. Abnormal expression or function of these channels can lead to uncontrolled cell division, a primary hallmark of cancer (Capatina et al., 2022; Leanza et al., 2016). Voltage gated Cl^−^ channels play a role in cell cycle regulation, cell proliferation, migration, and apoptosis in many cancer cells (Rao et al., 2015). However, not many groups have specifically investigated the expression and function of ligand-gated ion channel (LGIC) receptors in TNBC. The Cys-loop LGIC family of receptors are expressed as pentameric membrane-bound receptors of multiple subtypes which have unique pharmacological properties (Alexander et al., 2017). Moreover, amino acid transmitters such as GABA (gamma-amino butyric acid) which endogenously activate the GABA type A receptor (GABA_A_R) LGIC can also act as an energy source for cancer cells via direct conversion to TCA cycle intermediates (Ravasz et al., 2017). Activation of GABA_A_R mediates hyperpolarization via Cl^−^ ion influx in the adult central nervous system (CNS), promoting inhibitory neurotransmission (Knoflach et al., 2016). GABA_A_R have been shown to be expressed in many peripheral cancers (Bhattacharya et al., 2021). We recently showed that pharmacological antagonism and genetic knockdown of GABA_A_ β_3_ subunit decreases TNBC proliferation and migration via decreased cyclin D1 expression and increased p21 expression, combined with cell cycle arrest in the G_0_/G_1_ phase (Bundy et al., 2024). Similarly, the GABA_A_R π subunit is implicated in pancreatic cancer, and α3 GABA_A_R subunit shows overexpression in HER2+ breast cancer (Juvale et al., 2021; Gumireddy et al., 2016; Takehara et al., 2007). GABA_A_R π has been shown to stimulate breast cancer cell invasion through the ERK1/2 pathway (Sizemore et al., 2014) and it also interacts with EGFR and sustains EGFR expression in TNBC (Li et al., 2021). However, the functionality of such overexpressed GABA_A_R subunits in peripheral cancers outside the brain is understudied. With respect to brain cancers, benzodiazepine analogs have been shown to induce Cl^−^ efflux from the medulloblastoma cells, depolarizing their mitochondria and inducing fission (Kallay et al., 2019). Little is known about the cell-surface expression and functional status of the upregulated GABA_A_R in cancers outside the brain, i.e. if they mediate Cl^−^ ion flux. Understanding the mechanisms by which upregulated GABA_A_R function and mediate Cl^−^ flux in TNBC could identify new approaches to target TNBC, since ion flux (e.g., Ca^2+^, K^+^) can alter tumor growth and metastasis (Prevarskaya et al., 2018). Cl^−^ ions play a role in cell-cycle progression in gastric cancer; wherein low [Cl^−^] levels induce G_1_ cell cycle arrest and upregulation of p21 (Shiozaki et al., 2011). Therefore, we wanted to investigate whether GABA_A_R overexpressed in TNBC are expressed on the cell-surface and if they mediate Cl^−^ flux. Elucidating cell-surface expression is important since membrane-bound receptors are easier to target with small molecules and antibody-based approaches. Understanding the nature of GABA_A_R mediated Cl^−^ flux in TNBC can further help in mapping out how Cl^−^ flux may contribute to increased proliferation and migration in TNBC.

To address these questions, we employed a panel of TNBC and non-tumorigenic MCF-10A cell lines. We employed surface biotinylation experiments to study GABA_A_R localization and N-(Ethoxycarbonylmethyl)-6-methyl quinolinium bromide (MQAE) fluorescence quench assays to assess function and direction of GABA_A_R-mediated Cl^−^ flux. Our results indicate that α1 and β3 subunit containing GABA_A_R in TNBC cell lines are localized on the cell surface and are functionally active, mediatng GABA-mediated Cl^−^ influx. Further, employing TNBC cells with GABA_A_ β3 subunit knockdown, we show that GABA_A_R mediated Cl^−^ influx is attenuated after knockdown. Moreover, GABA_A_R antagonist bicuculline (BC) decreased cell proliferation in TNBC cells.

## Methods

### Cell culture

All cell lines were obtained from ATCC. MCF-10A cells were cultured in mammary epithelial basal medium (MEBM) (Lonza, MD) supplemented with 5% horse serum (Invitrogen), 20 ng/mL epidermal growth factor (Lonza), 0.5 mg/mL hydrocortisone (Lonza), 10 μg/mL insulin (Sigma-Aldrich, MO), 100 ng/mL cholera toxin (Sigma-Aldrich), and 1% penicillin/streptomycin (ThermoFisher Scientific, MA). MDAMB231 cells were cultured in Dulbecco’s modified eagle medium (DMEM) (ThermoFisher Scientific) supplemented with 10% heat-inactivated fetal bovine serum (FBS) (ThermoFisher Scientific), 1% sodium pyruvate (ThermoFisher Scientific), and 1% penicillin/streptomycin (ThermoFisher Scientific). BT-549 cells were cultured in RPMI-1640 growth medium (ATCC, VA) supplemented with 10% FBS, 0.023 IU/mL bovine insulin, and 1% penicillin/streptomycin. HCC1806 cells were cultured in RPMI-1640 growth medium supplemented with 10% FBS and 1% penicillin/streptomycin. All cell lines were incubated at 37°C in a 5% CO_2_ incubator.

### Cell proliferation MTS assay

2.5 × 10^4^ cells were plated on 96 well plates and incubated overnight at 37°C, 5% CO_2_. Cells were treated with GABA_A_R antagonist BC for 48 h to assess effects of pharmacological inhibition of GABA_A_R. 20 μL of CellTiter 96^®^ AQ_ueous_ One Solution Reagent containing a tetrazolium compound [3-(4,5-dimethylthiazol-2-yl)-5-(3-carboxymethoxyphenyl)-2-(4-sulfophenyl)-2H-tetrazolium, inner salt; MTS] (Promega, WI) was added to each well, incubated at 37°C, 5% CO_2_. Absorbance was read on a plate reader at 490 nm.

Lentiviral Mediated Knockdown of GABA_A_R subunit in TNBC Epithelial Cells: As described earlier (Bundy et al., 2024), to knockdown the GABA_A_ β3 subunit, TNBC cells (HCC1806 and BT 549) were cultured in appropriate complete medium until cells were 50% confluent. Medium was replaced with polybrene (5 μg/mL) containing medium to increase transduction efficiency. Cells were infected with transduction-ready scramble control shRNA lentiviral particles (#TR30021V, Origene, MD) or human GABA_A_R shRNA lentiviral particles (constructs # 2 and 3 targeting the GABA_A_ β_3_ gene, #TL304428V, Origene) for 24 h at multiplicity of infection (MOI) of 5. Constructs 2 and 3 were chosen out of 4 unique constructs since they led to the highest GABA_A_ β3 protein knockdown in HCC1806 and BT549 cells (Bundy et al., 2024). Stably transduced cells were selected with puromycin (1–2.5 μg/mL).

### Cell-surface biotinylation

The protocol followed was based on a cell surface protein biotinylation protocol we employed previously (Bundy et al., 2024). Briefly, MCF-10 A and TNBC cell lines were incubated with membrane-impermeable biotin, lysed with lysis buffer. Biotinylated proteins were pulled down with neutrAvidin Ultralink beads and analyzed via western blotting. GAPDH was used as a loading control and as a cytosolic marker, thus GAPDH signal was only detected in the ‘input’ and not ‘pulldown’ samples. ABCB1 expression, also known as multidrug resistance 1 (MDR1), was used as a positive control to confirm detection of a membranous protein.

### Western blotting

Western blotting was carried out as previously described (Bundy et al., 2024). Briefly, protein samples (20 μg) were separated by SDS-PAGE and transferred to a PVDF membrane for probing and blocked in TBS-Tween supplemented with 5% nonfat dry milk for 1 h at room temperature (RT). The membranes were incubated with these primary antibodies: GABA_A_ β3 (1:1,000, #73–149, RRID: AB_2109585, Antibodies Inc., CA), GABA_A_ ɑ1 (1:1,000, #75–136, RRID: AB_2108811, Antibodies Inc.), ABCB1 (ABCB1 (1:1,000, #12683, RRID: :AB_2715689 Cell Signaling) and Glyceraldehyde-3-phosphate dehydrogenase (GAPDH) (1:20,000, #10R-2932, RRID: AB_11199818, Fitzgerald, MA) antibody as the loading control. Each blot was incubated with the respective dilution of primary antibody overnight with the exception of GAPDH antibody which was incubated for 1 h. IRDye 680RD secondary antibodies (1:10,000, LI-COR BioSciences, NE) were used to visualize bound primary antibodies. The Odyssey CLx Imaging System (LI-COR BioSciences) was utilized for near-infrared fluorescent detection of proteins. Image Studio software on the Odyssey CLx was used to carry out densitometry analysis (LI-COR BioSciences).

### Preparation of buffer for MQAE fluorescence assays

We employed a Cl^−^free HEPES buffer containing 10 mM HEPES, 95 mM Sodium Nitrate, 2.5 mM Potassium Nitrate and 1.8 mM Calcium Nitrate since NO_3_^−^ in the range between 0 and 100 mM does not quench MQAE fluorescence (Hiroshi Kaneko et al., 2002; Rocha-Gonzalez et al., 2008). This complete HEPES buffer was then adjusted to pH of 7.4 with 0.1 M sodium hydroxide and osmolarity of 310 mOsm/kg and 1 M sucrose, respectively. For double ionophore calibration experiments, the K^+^/H^+^ antiporter nigericin (5 μM) was added to this HEPES buffer to remove H^+^ and OH^−^ gradients, and the Cl^−^/OH^−^antiporter tributyltin (10 μM) was added to equalize Cl^−^ gradients; i.e. to ensure that the intracellular [Cl^−^] was equal to the extracellular [Cl^−^]. The choice of antiporter concentrations was guided by previously published studies (Krapf et al., 1988; Hiraoka et al., 2010).

### MQAE loading

2.5 × 10^5^ cells/well were seeded into 24 well plates and left overnight to adhere. The next day, cells were washed with HEPES buffer three times. Cells were then loaded with 5 mM N-(Ethoxycarbonylmethyl)-6-methyl quinolinium bromide (MQAE), a halide-sensitive dye (ThermoFisher Scientific) in HEPES buffer for 90 min at 37°C and 5% CO_2_. Cells were washed again three times with HEPES buffer to remove extracellular MQAE and exposed to respective treatments and controls explained in the methods below (Cl^−^ calibration and GABA_A_R mediated Cl^−^ flux). Fluorescence of MQAE was read on a plate reader (BioTek Synergy Neo2 multimode microplate reader, ThermoFisher Scientific) at an excitation wavelength of 350 nm and emission wavelength of 460 nm.

### Cl^−^ calibration

After MQAE loading incubation and HEPES buffer washes, cells were exposed to various concentrations of potassium chloride (KCl) (0–80 mM) in HEPES buffer with double ionophores (5 μM nigericin and 10 μM tributyltin to equalize Cl^−^ gradients) to assess MQAE fluorescence quenching. The emitted MQAE fluorescence intensity is inversely related to the [Cl^−^] of the MQAE-containing solution due to quenching by a collisional mechanism with a linear relation as described by the Stern Volmer equation: F_0_/F_t_ = 1 + K_sv_ [Cl^−^] (Krapf et al., 1988).

### MQAE fluorescence assay to assess GABA_A_R-mediated intracellular Cl^−^ changes

Based on Cl^−^ calibration experiments, cells were loaded with MQAE and incubated with HEPES buffer with 60 mM KCl. Cells were exposed to various concentrations of GABA (0–3,000 μM) in HEPES buffer with 60 mM KCl to assess fluorescence quenching. Relative Cl^−^ flux was calculated by the Stern–Volmer equation, where F_0_ is the initial fluorescence before GABA treatment, and F_t_ is the final fluorescence after GABA treatment. EC_50_ values were determined by plotting a dose-response curve and interpolating the concentration at which the response reaches 50% of its maximum effect.

To assess pharmacological GABA_A_R inhibition of Cl^−^ flux, respective wells were pre-treated with GABA_A_R antagonist 10 μM BC for 15 min to block receptor activity before 100 μM GABA ligand was added to the respective wells. Dosing of BC was chosen based on the cell viability data (Figure 4). Next, to assess pharmacological positive allosteric modulation of GABA_A_R, 7 μM diazepam was added to respective wells simultaneously with 5 μM GABA. Dosing for BC, diazepam and GABA was chosen based on well-established prior research on GABA_A_R in the CNS field (Wongsamitkul et al., 2017). Fluorescence quenching was assessed via the plate reader, and relative Cl^−^ flux was calculated using the Stern–Volmer plots of fluorescent ratios versus concentration of the quencher, where F_0_ is the initial fluorescence before treatment, and F_t_ is the final fluorescence in the presence of the quencher (Motz et al., 2023).

### MQAE fluorescence assay to assess GABA_A_R-mediated intracellular Cl^−^ flux after GABA_A_ β_3_ subunit knockdown

HCC 1806 and BT 549 cells that have undergone GABA_A_ β_3_ lentiviral mediated knockdown as described previously were employed (Bundy et al., 2024). Knockdown cells (Scramble control, GABA_A_ β_3_ KD Constructs # 2 and 3) were washed with HEPES buffer, loaded with MQAE, washed with HEPES again, and then exposed to HEPES buffer with 60 mM KCl and various concentrations of GABA (100–1,000 uM). Fluorescence quenching was assessed as described above.

### Cell viability assay

Cell viability experiments were performed as described in our previous study (Bundy et al., 2024). Cells were treated with BC at various concentrations (0–300 uM) for 48 h. As compared to Bicuculline Methiodide (BCM), BC does not have a quaternary ammonium charge and therefore, it is blood brain barrier permeable. In previous experiments (Bundy et al., 2024), BCM was used due to its blood brain barrier impermeability, making BCM suitable for future *in vivo* studies. We chose BC here in these experiments since BC is the parent compound and has a higher affinity for GABA_A_R and is better suited to study pharmacological inhibition of GABA_A_R (Johnston, 2013). Absorbance of the CellTiter 96^®^ AQueous One Solution Reagent containing a tetrazolium compound [3-(4,5-dimethylthiazol-2-yl)-5-(3- carboxymethoxyphenyl)-2-(4-sulfophenyl)-2H-tetrazolium, inner salt; MTS] (Promega, WI) was read at 490 nm. IC_50_ values were determined by plotting a dose-response curve and interpolating the concentration at which the response is reduced by 50% compared to the control.

### Statistical analysis

t-test, one-way ANOVA followed by a Tukey post-hoc test in GraphPad Prism 8.0 (Boston, MA) were employed as needed. *p* < 0.05 was considered significant.

## Results

### Biotinylation suggests that β3 and α1 subunits of GABA_A_R are localized on the cell surface

**FIGURE 1:**
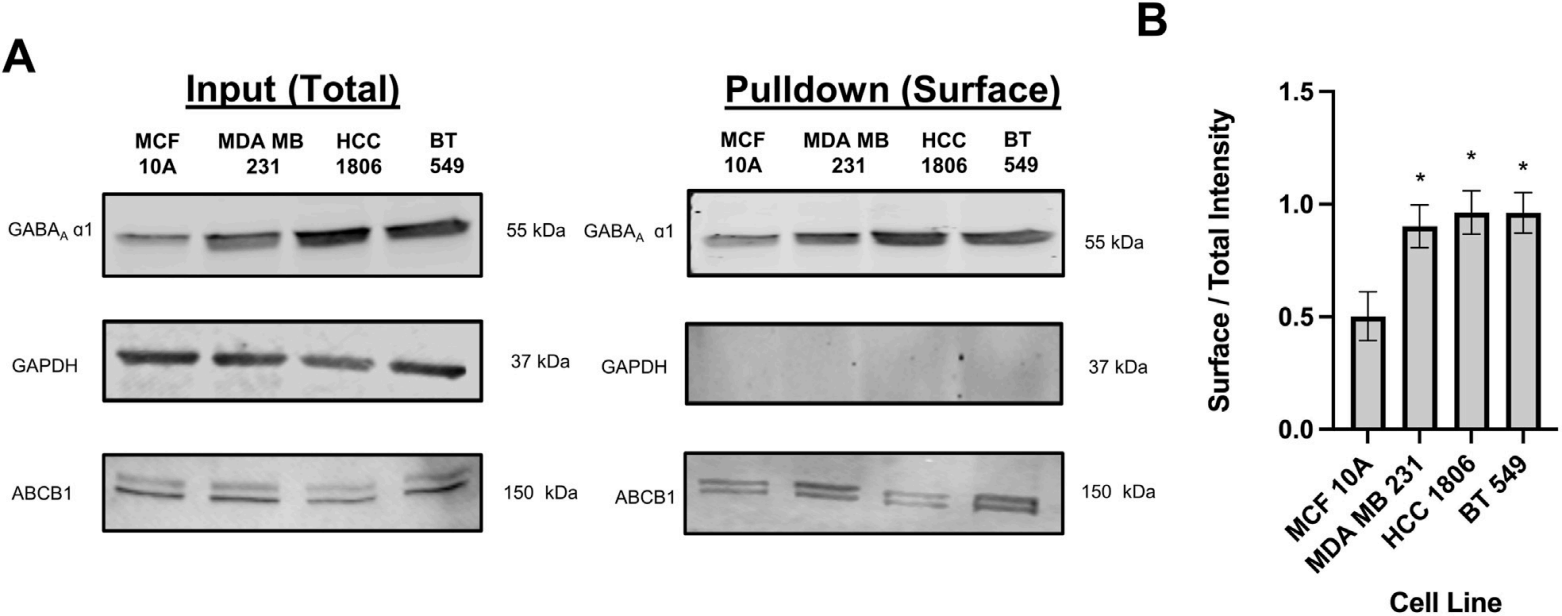
Biotinylated protein from TNBC cells and MCF10A cells show that GABA_A_R α1 subunit is localized on the cell surface. **(A)** GABA_A_ α1 subunit protein expression in whole cell samples (input) versus biotinylated samples (pulldown) in TNBC cells and MCF10A cells showing that GABA_A_R α1 subunits are localized on the cell surface, n = 3. GAPDH is a cytosolic marker shown as loading control. ABCB1 protein expression was used as a positive control to confirm the detection of a membrane-bound protein in the samples. **(B)** Corresponding densitometry of the ratio of pulldown to input samples across MCF10A and TNBC cell lines. * represent significance compared to MCF10A. Data are presented as mean±SE, *p < 0.05 (ANOVA).

Cell surface biotinylation assays were performed in MCF 10A cells and TNBC cells to confirm localization. Results confirm that the GABA_A_ β3 and α1 subunits are located on the cell surface in MCF10A cells and TNBC cells indicating that the GABA_A_R is membranous (Figure 1). Consistent with western blotting results with whole cell lysates reported by us, GABA_A_ α1 and β3 subunit expression is significantly higher in HCC 1806 and BT 549 cell lines as compared to MCF10A cell lines (Bundy et al., 2024).

### MQAE dye shows no significant change in fluorescence over time indicating no dye leakage during experiments

**FIGURE 2:**
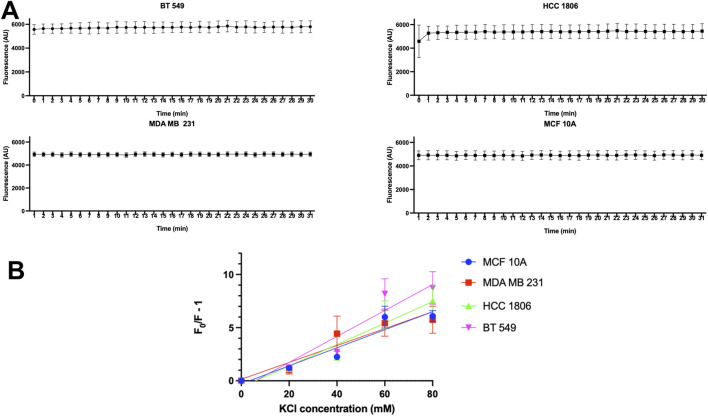
MQAE fluorescence intensity indicates no major dye leakage in TNBC cells and MCF10A cells and Stern–Volmer plots. **(A)** Fluorescent intensity of MQAE of MCF 10A, MDA MB 231, HCC 1806 and BT 549 cells over time, n = 3. **(B)** Stern–Volmer plots for MCF 10A, MDA MB 231, HCC 1806 and BT 549 cells indicate that the KCl concentration used as a constant in MQAE Cl-flux experiments is 60 mM, n = 3. Data are presented as mean±SE (ANOVA).

Dye leakage experiments were done to test whether MQAE dye leakage was a contributing factor in the change in fluorescence. Results show that there is no detectable change in fluorescence over 30 min of time in MCF 10A cells and TNBC cells indicating that no detectable dye leakage occurs during this time and therefore leakage does not contribute to fluorescence changes in MQAE experiments (Figure 2A).

### Relationship between MQAE fluorescence quench and [Cl^−^]_i_ in TNBC and MCF10A cells

To convert the fluorescence intensity into the [Cl^−^]_i_, the relationship between intracellular MQAE fluorescence and [Cl^−^]_i_ was determined using the double ionophore technique. MCF10A and TNBC cells were exposed to various concentrations of KCl (0–80 mM) as the quencher of MQAE fluorescence. The Stern–Volmer plot showing the quench in fluorescence intensity against KCl concentrations is shown in Figure 2B, indicating a K_SV_ of 5.56 M^-1^, 6.77 M^-1^, 7.32 M^-1^, 8.56 M^-1^ for MCF 10A, MDA MB 231, HCC 1806, and BT 549 cells, respectively. These K_sv_ values reflect Cl^−^sensitivity of MQAE and fall within the range of K_sv_ values (5–25 M^-1)^ in previous studies with MQAE in neurons (Weilinger et al., 2022; Kaneko et al., 2004).

### MCF 10A cells and TNBC cells exposed to GABA ligand show a concentration-dependent increase in Cl^−^ ion influx

**FIGURE 3:**
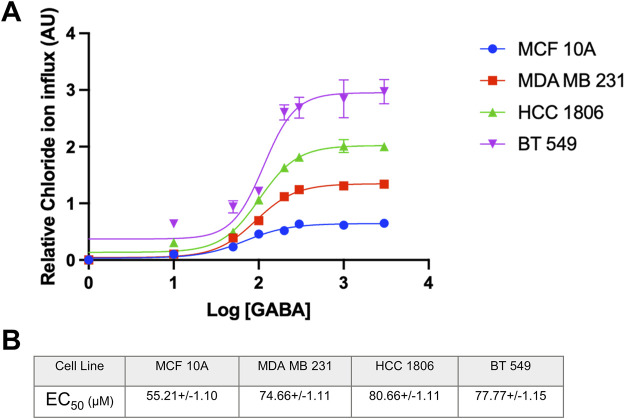
EC_50_ of GABA ligand concentration and relative Cl-concentration in TNBC cells and MCF 10A cells. **(A)** EC_50_ curve of GABA ligand concentration on MCF 10A, MDA MB 231, HCC 1806, and BT549 cells n = 3. **(B)** EC50 values of GABA (µM) interpolated from the curve for each cell line, respectively. Data are presented as mean±SE (ANOVA).

Non-tumorigenic MCF 10A cells and TNBC cells were exposed to increasing concentrations of GABA to assess GABA_A_R function and the direction of ion flux when activating the receptor. Results indicate a significantly higher quench in fluorescence in TNBC cells as compared to MCF10A cells indicating that the GABA_A_R is functional and expressed at a higher level in TNBC cells. Additionally, there is an increase in intracellular Cl^−^ concentration in all cell lines, indicating that the directionality of Cl^−^ ions in the GABA_A_R is influx under these experimental conditions (Figure 3A). As shown by GABA dose-response curves, relative Cl^−^ influx efficacy shows the rank of BT549>HCC1806>MDA MB231>MCF10A cells. These data are in agreement with surface biotinylation data where GABA_A_ α1 and β3 show the highest expression in BT549 and HCC1806 cells as compared to MCF10A cells. Calculated EC_50_ values for GABA in MCF10A and 3 TNBC cell lines are shown in Figure 3B.

### GABA_A_R-mediated intracellular Cl^−^ flux is pharmacologically modulated by GABA_A_R ligands

**FIGURE 4:**
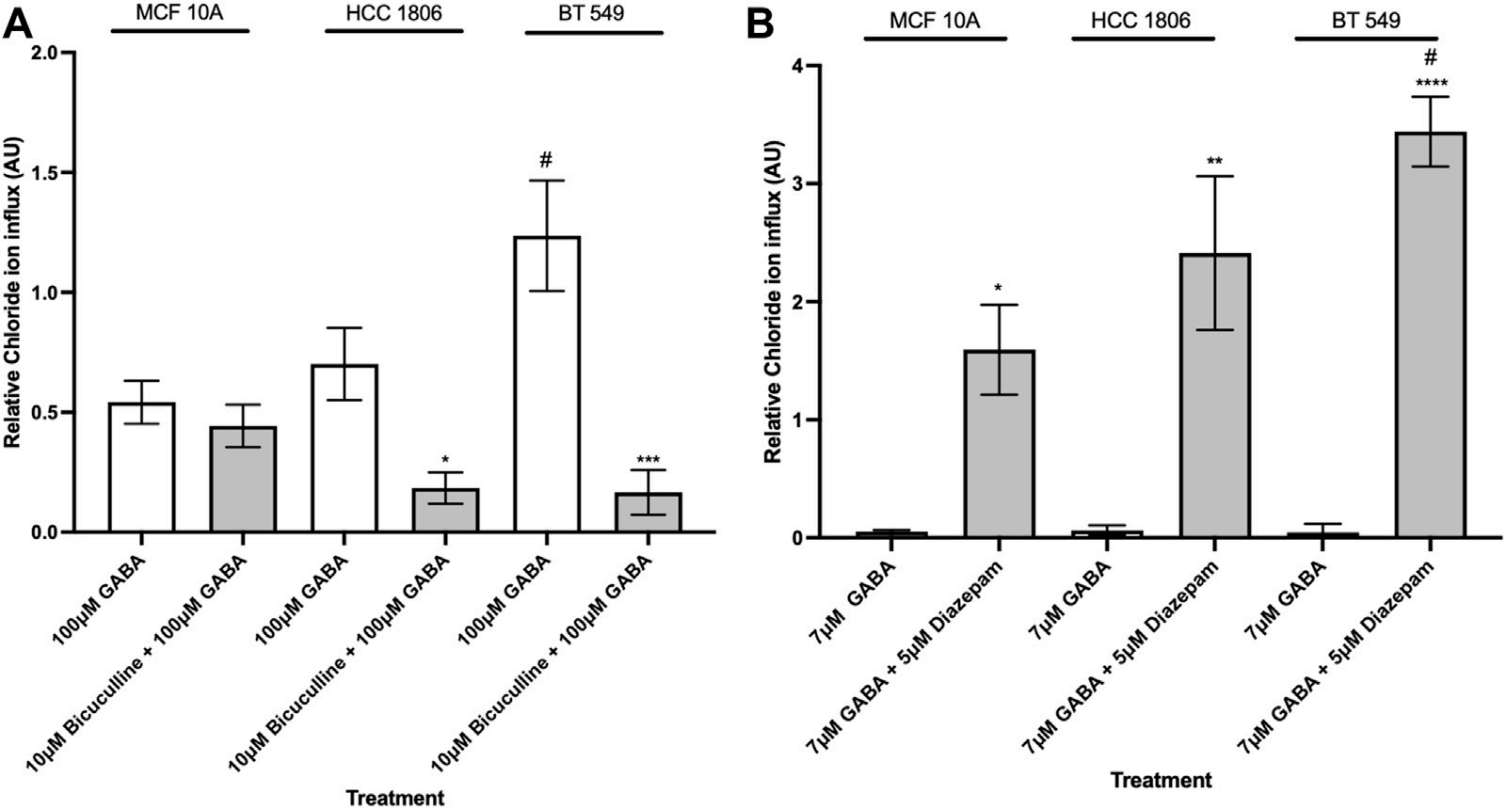
Intracellular Cl^−^ in TNBC cells and MCF 10A cells decreases when exposed to GABA_A_R competitive antagonist and increases when exposed to GABA_A_R positive allosteric modulator. **(A)** Relative Cl^−^ in TNBC cells and MCF 10A cells exposed to 100 µM GABA alone and in combination with 10 µM GABA_A_R competitive antagonist, Bicuculline, n = 3. **(B)** Relative Cl^−^ in TNBC cells and MCF 10A cells exposed to 7 µM GABA alone and in combination with 5 µM GABA_A_R positive allosteric modulator, Diazepam, n = 3. * represent significance compared to 7 µM GABA alone within each cell line, respectively. # represent significance compared to the HCC 1806 cell line. Data are presented as mean±SE, *p < 0.05, **p < 0.01, ***p < 0.001,****p < 0.0001 (ANOVA).

In order to characterize pharmacological properties, MCF10A and TNBC cells were exposed to well-established GABA_A_R modulators-the antagonist BC, and the positive allosteric modulator, Diazepam. When exposed to BC, Cl^−^ influx decreased in BT 549 and HCC 1806 cells and showed no significant change in MCF 10A cells (Figure 4A). When exposed to Diazepam, MCF 10A cells and TNBC cells resulted in an increase in Cl^−^ influx (Figure 4B). Moreover, BT 549 cells showed a significantly higher level of intracellular Cl^−^ than MCF 10A and HCC 1806 cells.

### GABA mediated Cl^−^ influx is attenuated in GABA_A_β_3_ subunit knockdown cells

**FIGURE 5:**
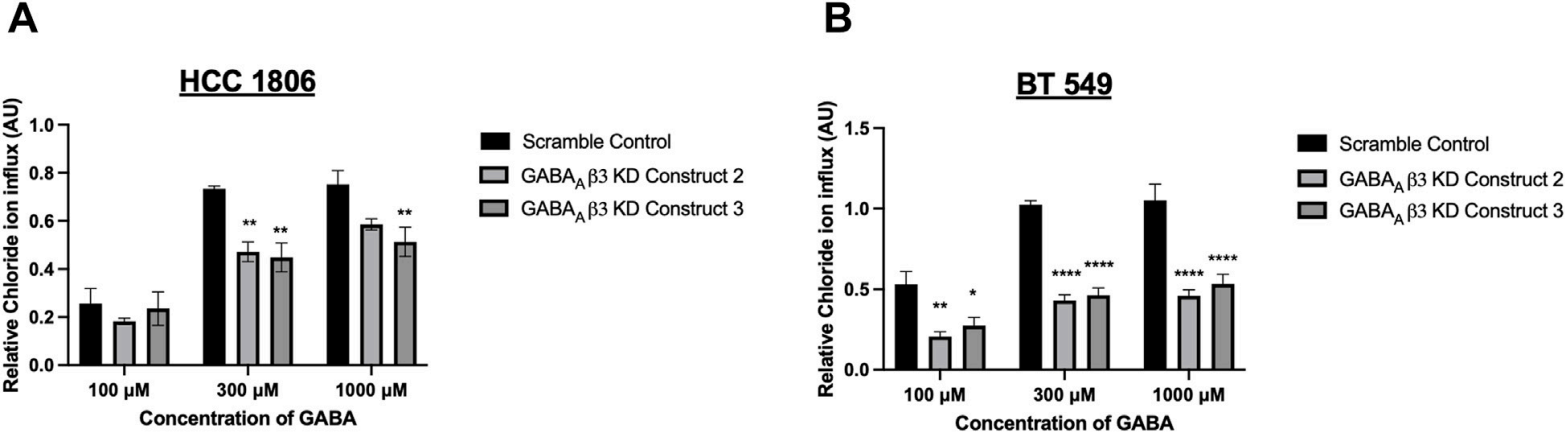
Relative GABA_A_R-mediated Cl^−^ flux in TNBC cells decreases after GABA_A_β_3_ subunit knockdown. **(A)** Relative Cl^−^ in HCC 1806 cells that have undergone GABA_A_β_3_ subunit knockdown (scramble and KD constructs 2,3) exposed to 100 μM, 300 μM, and 1,000 μM GABA, n = 3. **(B)** Relative Cl^−^ in HCC 1806 cells that have undergone GABA_A_β_3_ subunit knockdown (scramble and KD constructs 2,3) exposed to 100 μM, 300 μM, and 1,000 μM GABA, n = 3. * represent significance compared to the scramble control within concentration of GABA, respectively. Data are presented as mean±SE, *p < 0.05, **p < 0.01, ****p < 0.0001 (ANOVA).

TNBC cells that have undergone GABA_A_β_3_ subunit knockdown were exposed to GABA at various concentrations. HCC 1806 cells with GABA_A_β_3_ subunit knockdown show a significant decrease in relative intracellular Cl^−^ when exposed to 300 μM and 1,000 µM GABA (Figure 5A). BT 549 cells with GABA_A_β_3_ subunit knockdown also show a significant decrease in relative intracellular Cl^−^ when exposed to 100 μM, 300 μM, 1,000 µM GABA (Figure 5B). In addition, in BT 549 cells, Construct 2 and 3 show a more significant reduction of intracellular Cl^−^ than HCC 1806 knockdown cells.

### GABA_A_R antagonist BC significantly decreases cell viability in TNBC cells as compared to MCF 10A cells

**FIGURE 6:**
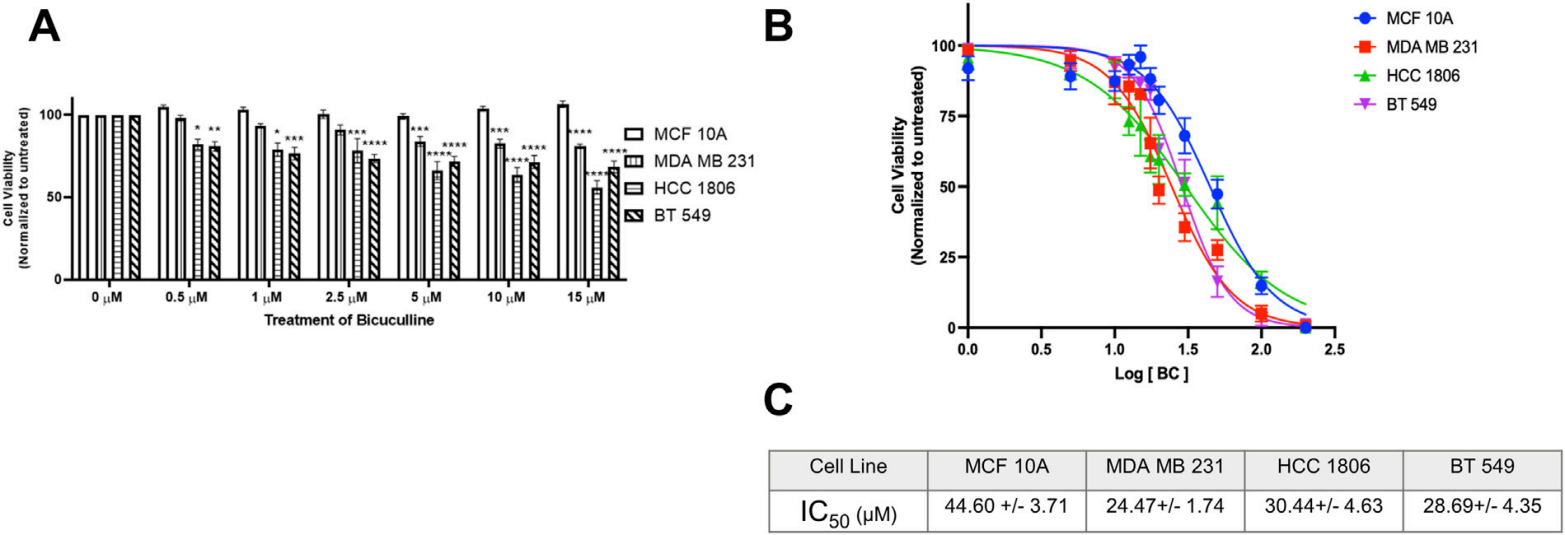
GABA_A_R competitive antagonist Bicuculline decreases cell proliferation in TNBC cells. **(A)** Cell viability of MCF 10A cells and TNBC cell lines exposed to Bicuculline for 48 h at various concentrations, n = 3. **(B)** Complete IC_50_ curve for Bicuculline in all TNBC cell lines **(C)** IC_50_ values of Bicuculline for all TNBC cell lines * represent significance compared to MCF10A within each concentration, respectively. Data are presented as mean±SE, *p < 0.05, **p < 0.01, ***p < 0.001, ****p < 0.0001 (ANOVA).

Non-tumorigenic MCF 10A cells and TNBC cells were exposed to GABA_A_R antagonist BC at various concentrations. Results indicate that there is a significant decrease in cell viability in all TNBC cell lines at 5 µM (Figure 6A) as compared to MCF10A cells. IC_50_ values suggest that TNBC cell lines are more sensitive to BC as compared to MCF 10A cells (Figures 6B,C). Morphology of the cells exposed to BC are in Supplementary Section.

## Discussion

Our previous study showed that GABA_A_ β3 subunit plays a vital role in proliferation, migration and cell-cycle progression of TNBC cells (Bundy et al., 2024). To elucidate the mechanisms by which β3 subunit containing GABA_A_R mediate these effects in TNBC and to develop therapeutic approaches to target these GABA_A_R, confirming surface localization of GABA_A_R is imperative to deem the receptor as druggable in TNBC cells. Surface biotinylation experiments reported here confirm that GABA_A_ α1 and β3 subunits, which are critical for forming the GABA-binding interface, are localized at the cell surface. We next investigated if these cell-surface GABA_A_R are functional and whether they mediate Cl^−^ influx or efflux in TNBC cells. In the adult CNS, binding of GABA to GABA_A_R causes a conformational change, opening the ion channel, allowing Cl^−^ ions to flow through (Lynagh and Pless, 2014). Our results suggest that GABA causes a concentration-dependent increase in Cl^−^ influx leading to a fluorescence quench. This GABA_A_R-dependent Cl^−^ influx occurs to a much greater degree in TNBC cells as compared to MCF10A cells. This functional rank efficacy for GABA observed here in TNBC vs. MCF10A cells also correlates with the rank order of α1 and β3 GABA_A_ total protein levels (BT549>HCC1806>MDA MB231>MCF 10A) reported earlier by us (Bundy et al., 2024). The EC_50_ values for GABA obtained from our experiments are within the EC_50_ range of 6–106 μM established in literature from various expression systems (Hadingham et al., 1993; Karim et al., 2013; Baur and Sigel, 2003).

We next studied the modulation of GABA_A_R in cell lines using well-characterized GABA_A_R pharmacological modulators such as BC (GABA_A_R competitive antagonist) and Diazepam (GABA_A_R positive allosteric modulator). Diazepam is a classic benzodiazepine that binds to allosteric site at the α-γ interface of the GABA_A_R, causes a conformational change, enhancing GABA-mediated channel opening frequency and Cl^−^ influx (Olsen, 2018). The competitive GABA_A_R antagonist, BC, binds to the α-β interface and inhibits the receptor, therefore inhibiting flow of Cl^−^ ions (Johnston, 2013). The GABA_A_R-mediated Cl^−^ influx was inhibited by BC and potentiated by diazepam, further supporting GABA_A_R function.

It is important to note that even though GABA_A_R activation typically results in Cl^−^ influx in the adult CNS, this is not always the case, especially in brain cancers. For example, the α5 GABA_A_ subunit is overexpressed in medulloblastoma, however the GABA_A_R in medulloblastoma show efflux of Cl^−^ ions, contributing to mitochondrial depolarization, inducing mitochondrial fission and dysfunction (Kallay et al., 2019). On the other hand, little is known about GABA_A_R subunit composition and function in peripheral cancers where GABA_A_R overexpression has been detected (with α3, π GABA_A_R subunits). Many studies indicate the overexpression/knockdown of specific GABA_A_R subunits can affect cancer cells, but these studies do not investigate if these subunits form functional receptors (Gumireddy et al., 2016; Sizemore et al., 2014). Here, we show that in contrast to GABA_A_R in medulloblastoma, GABA_A_R overexpressed in TNBC cells mediate Cl^−^ influx to a much higher extent than MCF10A cells. These observations allow us to further study how Cl^−^ influx affects proliferation and migration of TNBC cells. These findings are also supported by results from TNBC cells that have undergone GABA_A_β3 subunit knockdown which show a reduction in GABA_A_R-mediated Cl^−^ influx as compared to cells treated with scramble control.

With respect to Cl^−^ ions, intracellular Cl^−^ accumulation via Na^+^, K^+^, 2Cl^-^ (NKCC) cotransporter activity is implicated in glioma cells (Luo et al., 2020). It is also known that low intracellular Cl^−^ can induce cell cycle arrest in the G1 phase in prostate cancer (Hiraoka et al., 2010). Additionally, several studies suggest that membrane hyperpolarization at the G1/S checkpoint is required for S phase initiation. For example, depolarizing the cell membrane halts G1/S progression in MCF-7 breast cells (Wonderlin et al., 1995). Therefore, we speculate that if Cl^−^ ion influx is blocked via GABA_A_R inhibition or genetic knockdown, it could relatively depolarize the membrane potential of the TNBC cells, and halt cell cycle progression. Thus, our results show, for the first time, that GABA_A_R overexpressed in TNBC mediate Cl^−^ influx. Moreover, pharmacological inhibition of this Cl^−^ influx with BC also decreases TNBC cell viability, with TNBC cells showing a higher sensitivity to BC as compared to MCF10A cells. Therefore, decreasing intracellular Cl^−^ may be a novel mechanism by which TNBC proliferation and migration can be controlled. Future studies will focus on employing patch clamp electrophysiology to elucidate the membrane potential, E_Cl_ (Chloride reversal potential) and Cl^−^ influx kinetics in individual TNBC cells after GABA_A_R modulation. These studies will further guide the design of novel ligands that can target this membrane-bound LGIC that is upregulated in TNBC.

## Data Availability

The original contributions presented in the study are included in the article/Supplementary Material, further inquiries can be directed to the corresponding author.
