# Supplementary figures and images for: GABA Type A receptors expressed in triple negative breast cancer cells mediate chloride ion flux
Source: Front Pharmacol. 2024 Oct 14;15:1449256. doi: 10.3389/fphar.2024.1449256 (PMC11513581; doi:10.3389/fphar.2024.1449256)

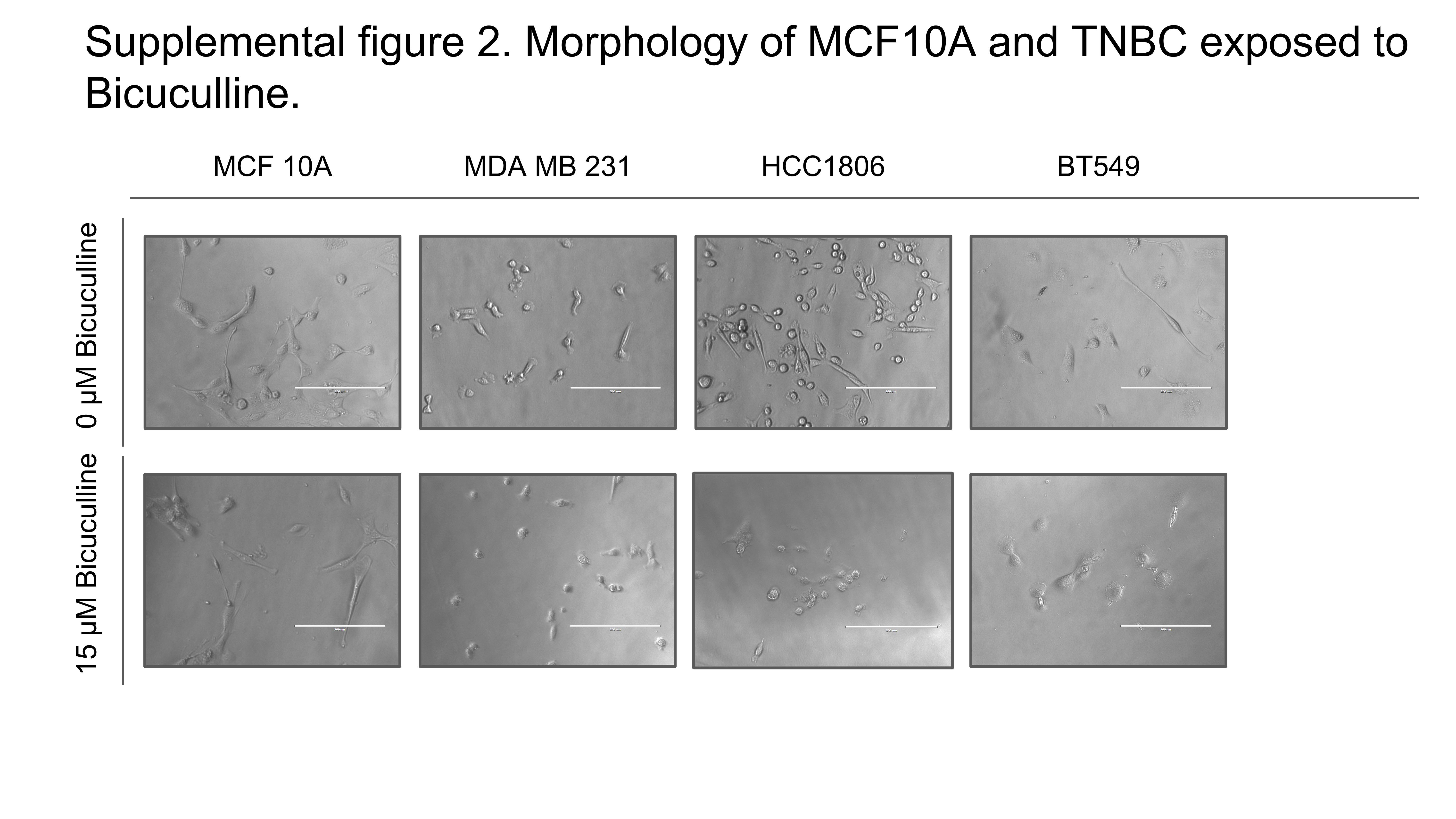

Supplement: Supplementary file 1 [file Image2.TIF]

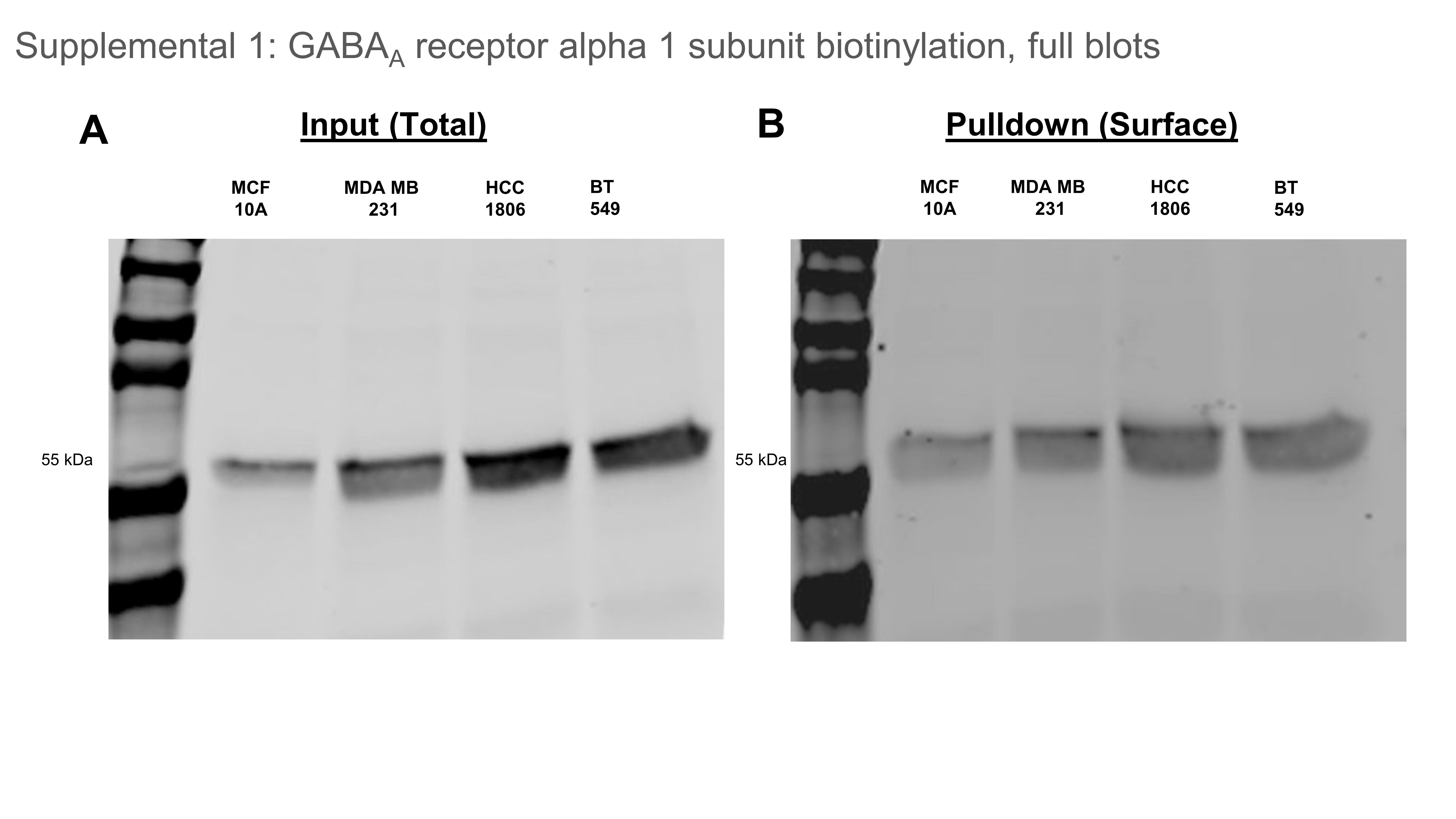

Supplement: Supplementary file 2 [file Image1.TIF]
